# Multimodal Imaging for Monitoring of Disease Progression in Cardiac Amyloidosis: Advances and Gaps in Evidence

**DOI:** 10.3390/jcdd13040152

**Published:** 2026-03-30

**Authors:** Claudia Meier, Roja Soutodeh, Stephan Gielen

**Affiliations:** 1 Klinikum Lippe, Universitätsklinikum Ostwestfalen-Lippe, Universitätsklinik für Kardiologie, Angiologie und Internistische Intensivmedizin, Campus Klinikum Lippe, 32760 Detmold, Germany; 2 Medical Faculty, Bielefeld University, 33615 Bielefeld, Germany

**Keywords:** cardiac amyloidosis, transthyretin amyloidosis, light-chain amyloidosis, echocardiography, bone scintigraphy, cardiac magnetic resonance imaging, multimodal imaging, disease progression

## Abstract

Among cardiac storage diseases, amyloidosis has emerged as a common cause of heart failure (HF), particularly in older people: it is diagnosed in up to 13–19% of patients with heart failure and preserved ejection fraction. Current treatments for transthyretin amyloidosis (ATTR) focus on stopping the misfolding of the TTR protein or reducing TTR production and treating the symptoms with cardiac medication, while systemic chemotherapy is the focus for light-chain amyloidosis (AL). New fibril clearance agents and gene therapies are currently in development. In addition to clinical and laboratory observations, multimodal imaging is essential for the monitoring of the effects of treatment on the progression of heart disease, but it is not yet included in established staging systems. This narrative review collects current multimodal imaging parameters that have been evaluated in clinical trials to assess the progression of cardiac amyloidosis and used in phase III intervention studies. These evolving findings are compared with current consensus recommendations to identify gaps in knowledge for specific imaging modalities, particularly cardiac MRI. Ultimately, the goal should be to standardize imaging of disease progression in cardiac amyloidosis so that the therapeutic effects of new pharmacological treatment options can be compared with the current standard of care.

## 1. Introduction

Cardiac amyloidosis (CA) represents a heterogeneous disease entity with a variable cardiac phenotype caused by amyloid fibril deposition in the myocardium, leading to restrictive cardiomyopathy. The two main types are immunoglobulin light-chain (AL) and transthyretin (ATTR) amyloidosis.

AL results from a clonal plasma cell disorder producing misfolded light chains that form amyloid fibrils, affecting the heart in up to 75% of cases. These fibrils exert direct cardiotoxic effects, making early diagnosis and treatment essential. Therapy involves cytotoxic chemotherapy to suppress amyloidogenic light chains, often followed by stem cell transplantation, achieving median survival beyond 4 years [1,2].

ATTR has its underlying cause in a misfolding of the liver-derived transthyretin (TTR) protein, a tetramer that normally transports thyroxine and retinol. It occurs as wild-type (ATTRwt)—an age-related form predominantly in men causing restrictive cardiomyopathy—or hereditary (ATTRv) amyloidosis due to TTR gene mutations, often presenting with both neuropathy and cardiomyopathy. It has emerged as the most frequent form of cardiac amyloidosis and as a common cause of heart failure (HF), particularly among elderly populations. It is diagnosed in 13–19% of patients with heart failure and preserved ejection fraction. Autopsy studies suggest that 25% of people over 80 years and 32% of patients >75 years with heart failure with preserved ejection fraction (HFpEF) are affected by ATTR [3]. Findings from the Transthyretin Amyloid Outcome Survey (THAOS) indicate a two-year mortality rate exceeding 30% for patients with wild-type ATTR and over 40% for those with the p.Val142Ile variant of hereditary ATTR [4], with cardiac involvement being the primary driver of mortality. Current pharmacological treatments aim to stabilize the TTR tetramer, silence TTR gene expression, or use emerging gene-editing therapies to reduce TTR production [2,5,6].

Advances in multimodal cardiac imaging now enable better diagnosis, treatment monitoring, and prognostic assessment [1,5,7,8,9,10]. A non-invasive diagnostic imaging pathway for suspected CA relies on echocardiography, bone scintigraphy and cardiac magnetic resonance imaging (CMR). Echocardiographic red flags include increased LV wall thickness, a “speckled” myocardium, apical sparing in strain reduction, and diastolic dysfunction. CMR typically shows increased left ventricular mass, elevated native T1 values, diffuse subendocardial or transmural late gadolinium enhancement (LGE), and raised extracellular volume (ECV). ATTR-CA can be diagnosed non-invasively when tracer uptake in bone scintigraphy is grade 2/3 and serum free light chains and immunofixation are normal. In contrast, in patients with plasma-cell dyscrasia and cardiac tracer uptake, cardiac biopsy is usually needed to confirm the amyloid type.

With the increasing use of expensive stabilizers like Tafamidis and Acoramidis, as well as—more recently—gene silencers like Patisiran, Vutrisiran, and Eplontersen, monitoring of the progression/regression of cardiac amyloidosis has become an essential component of a comprehensive treatment concept.

Surprisingly, a standard for multimodal imaging in follow-up has not yet been established, although some proposals have been published. Even the latest ACC clinical guideline [11], which was published recently, states that the question of how to monitor disease progression and response to therapy remains unanswered. Therefore, this narrative review aims

- To provide an overview of available imaging methods, their specific strengths and weaknesses in diagnosing amyloidosis, and their use in establishing a primary CA diagnosis and patient monitoring;
- To summarize the use of imaging for the monitoring/quantification of CA disease progression in phase III clinical trials on TTR stabilizers (i.e., imaging method and time intervals for monitoring);
- To propose an evidence-based scheme for multimodal imaging for evaluation of CA disease progression.

## 2. Overview of Multimodal Imaging in Cardiac Amyloidosis

Echocardiography is the undisputed number-one imaging tool for all heart diseases due to its widespread availability and relatively simple and rapid application. However, the diagnosis of cardiac involvement in amyloidosis cannot be confirmed with certainty. Preferred imaging strategies after echocardiography vary according to the research group’s disciplinary focus, ranging from nuclear medicine approaches, which are currently considered the non-invasive gold standard, to CMR. Compared to endomyocardial biopsy as a gold standard, CMR achieved a sensitivity of 85.7% and specificity of 92.0%, while nuclear scintigraphy showed 88.4% sensitivity and 87.2% specificity in a large meta-analysis [12]. The question of which method can better differentiate between AL and ATTR is also the subject of contradictory and controversial debate. All imaging parameters should be combined with electrocardiographic, clinical, biomarker, and other imaging findings to maximize diagnostic accuracy.

In addition to all the laboratory and imaging parameters, it should be noted that amyloidosis markedly impairs patients’ quality of life (QoL). Disease-specific QoL assessment tools are lacking. The ITALY study (Impact of Transthyretin Amyloidosis on Life Quality) developed a QoL assessment that could improve the characterization of the baseline, the monitoring of progression and the response to treatment and could have value in patient management and as a surrogate endpoint in clinical trials [13].

In 2021, the ASNC/AHA/ASE/EANM/HFSA/ISA/SCMR/SNMMI expert consensus published the most comprehensive recommendation to date for “Multimodality Imaging in Cardiac Amyloidosis” in two parts [14,15] to standardize image acquisition, interpretation, and reporting.

### 2.1. Echocardiography

#### 2.1.1. Echocardiography for Primary Diagnosis

**Figure 1:**
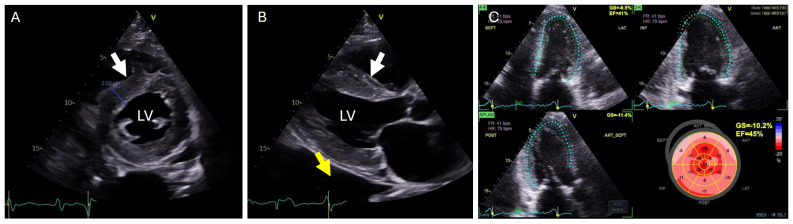
Typical echocardiographic findings in amyloidosis. (**A**) Parasternal short-axis view. (**B**) Parasternal long-axis view. (**C**) Three-dimensional apical long-axis view, along with strain measurement with reduced longitudinal strain and typical bullseye plot with “apical sparring”. White arrow: septal thickening up to 2 cm. Yellow arrow: pleural effusion. LV: left ventricle.

Cardiac amyloidosis is characterized by thickening of the myocardium, which is caused by the deposition of amyloid in the interstitium (extracellular space), as shown in Figure 1. Thickening can also be caused by increased left ventricular (LV) pressure due to other factors, such as aortic stenosis, or by other specific cardiomyopathies, such as hypertrophic cardiomyopathy. A left ventricular wall thickness >12 mm should raise suspicion of cardiac amyloidosis if no other cause for hypertrophy is present and/or additional clinical indicators point to amyloidosis. Thickening of the valves, interatrial septum, atrial walls, and right ventricular wall (>5 mm) is also typical. In two-dimensional echocardiography, the myocardium often appears hyperintense, described as a “salt-and-pepper” or “granular sparkling” pattern. The systolic function, measured as the left ventricular ejection fraction (LVEF), generally remains preserved until advanced disease stages, as it does not adequately reflect early longitudinal dysfunction. Using speckle-tracking analysis to assess global longitudinal strain (GLS), a characteristic “apical sparing” or “cherry-on-the-top” pattern can sometimes be identified, preserved apical strain relative to reduced basal and mid-ventricular strain [14]. While not pathognomonic, this is highly specific for cardiac amyloidosis and provides incremental diagnostic and prognostic value beyond other echocardiographic parameters. LGS shows a base-to-apex gradient. The pronounced regional LGS differences in cardiac amyloidosis are best explained by segmental variation in total amyloid burden rather than differences in the relative proportion of amyloid deposition [16]. Apical sparing likely reflects more than regional amyloid burden: factors such as selective fiber involvement and greater basal apoptosis/remodeling may contribute, with their relative impact varying with disease stage, amyloid type (AL vs. ATTR), patient factors, and comorbidities [17,18]. Several groups have shown that improvement in GLS after treatment is associated with a significant survival benefit in AL amyloidosis [19,20]. It has also been reported that the stroke volume index (Svi: stoke volume per heartbeat normalized to body surface area) achieves prognostic performance similar to that of LV strain in predicting survival. Atrial enlargement results from both the restrictive nature of the ventricular myocardium with predominant diastolic dysfunction and direct amyloid infiltration of the atrial walls. LA strain is markedly reduced, is related to amyloid burden, predicts AF and mortality, and adds prognostic value beyond established staging systems, even in early stages of disease. Reduced left atrial contractility increases the risk of thrombus and systemic embolism, even in sinus rhythm [21]. Adverse prognosis is linked to echo markers of restrictive LV filling (e.g., short mitral inflow deceleration time), and the noninvasive LA stiffness index may add prognostic value and improve early risk stratification beyond LA reservoir strain alone [22,23]. Assessment of diastolic dysfunction should include evaluation of the E/A ratio, the E/e′ ratio using tissue Doppler echocardiography, and pulmonary venous flow in the right upper pulmonary vein. With increasing disease severity, circumferential, hemodynamically insignificant pericardial effusion (especially in AL amyloidosis) and pleural effusions are common.

A two scenario-specific echocardiographic scoring system has been proposed for AL amyloidosis (A) to confirm or exclude cardiac involvement in patients with systemic AL amyloidosis and (B) to confirm or exclude cardiac amyloidosis in patients with increased LV wall thickness [24,25]. However, no echocardiographic scoring system with valid, reliable and universally applicable parameters has yet been established.

In the consensus document of Dorbala et al. [14,15], echocardiographic findings are summarized in three categories:

1. Not suggestive: Normal LV wall thickness, normal LV mass, normal atrial size, and septal or lateral tissue Doppler e’ velocity >10 cm/s;
2. Strongly suggestive: Increased LV wall thickness, increased LV mass, typical LV longitudinal strain pattern, mitral annular TDI < 5 cm/s, biatrial enlargement, small A wave in sinus rhythm, small pericardial and or pleural effusions;
3. Equivocal: Findings not described above.

Further diagnostic testing is indicated in the event of strongly suggestive and equivocal echocardiographic findings and clinical signs of amyloidosis. The recommended pathway for the diagnostic work-up of suspected cardiac amyloidosis, taking into account clinical and laboratory parameters, is described in detail in other sources [8,14,15,26].

#### 2.1.2. Echocardiography for Monitoring of Disease Progression

**Figure 2:**
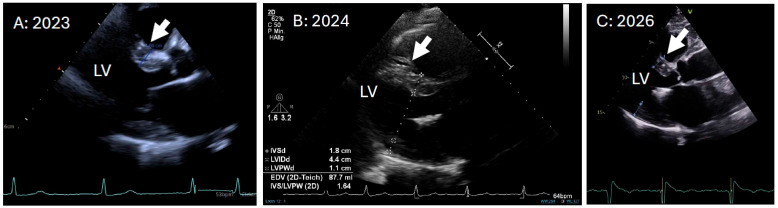
Septal thickening for disease monitoring in echocardiography. Although septal thickness is of limited use, it is recommended for monitoring and can indicate slow changes over time. Attention should be paid to minor measurement-related inaccuracies. (**A**) 2023 parasternal long axis before diagnosis (19 mm). (**B**) 2024 parasternal long axis after diagnosis and before start of therapy (18 mm). (**C**) 2026 parasternal long axis 2 years after start of therapy (15 mm). White arrow: septal thickening. LV: left ventricle.

A ≥2 mm increase in left ventricular wall thickness, as observed in approximately 8% of patients in clinical trials [27], is considered indicative of disease progression, whereas lesser changes likely reflect measurement variability. Because wall thickening evolves slowly and may be confounded by pressure increases due to various causes, it provides limited utility for short-term management (Figure 2). Routine echocardiographic evaluation of LV systolic function is recommended, with a ≥5% decline in ejection fraction or ≥5 mL reduction in stroke volume signifying progression. Reduced GLS and a reduced stoke volume (SV) index were found to be independently predictive of poor prognosis [28], and reduced myocardial fraction was found to be superior to LVEF in predicting prognosis [29] in ATTR-CA. As mentioned above, in AL-CA, GLS was also predictive of survival in several observations [30,31]. A meaningful change in GLS was determined as >−2% [30]. Garcia-Pavia et al. proposed a ≥1% worsening to indicate disease progression [6]. However, inter- and intra-individual measurement inaccuracies should be taken into account here. The myocardial contraction index, defined as the ratio of stroke volume to myocardial volume, provides a volumetric measure of myocardial shortening analogous to strain and serves as a prognostic marker superior to the ejection fraction [29]. RV dilatation on echo was associated with reduced survival [32], with findings published as early as 1997. Diastolic function should be assessed serially, recognizing its dependence on preload, heart rate, and rhythm. Early disease is typically characterized by abnormal LV strain and mild diastolic dysfunction, whereas advanced stages demonstrate reduced systolic performance, increased LV mass, and wall thickening.

Taking into account the positive therapeutic effect of tafamidis, there was a reduced rate of reduction in the SV index at 30 months [33] and slower rates of decline in the GLS and myocardial work index at 12 months [34] in ATTR-CA. It should be noted that this is an absolute deterioration in the parameters, even under treatment; therefore, interpretation can be challenging. The recently published largest post-approval, real-world dataset for ATTR-CM showed no significant differences in imaging parameters between patients on tafamidis and those not treated [35] after the first year. On the other hand, patisiran-treated patients showed improved GLS, reduced LVEDD and reduced mean LV wall thickness (an absolute improvement) compared with controls at 18 months [27].

The consensus group of Garcia-Pavia et al. [6] recommends echocardiography as the preferred imaging method for monitoring of ATTR-CA, with follow-up examinations every 6–12 months. This preference is driven by cost, availability and reliable long-term data from other studies.

### 2.2. Bone Scintigraphy

#### 2.2.1. Bone Scintigraphy for Primary Diagnosis

Bone scintigraphy is a key noninvasive tool for diagnosing CA. Common tracers include 99mTc-pyrophospate (PYP), 99mTc-3,3-diphosphono-1,2-propanodicarboxylic acid (DPD) and 99mTc-hydroxymethylene diphosphonate (HMDP) in Europe. To the best of our knowledge, there have been no studies comparing these radiotracers with each other until now. After excluding plasma cell dyscrasia, radionuclide scans can confirm ATTR-CA with >98% accuracy [26]. Of note, the reported high sensitivity and specificity largely come from expert centers and patients with advanced disease. The performance of 99mTc-PYP/DPD/HMDP scintigraphy in earlier or pre-clinical stages remains mostly unproven. DPD scintigraphy shows reduced sensitivity in early-onset Val50Met (formerly Val30Met) and rare variants such as Phe64Leu, indicating genotype-dependent diagnostic accuracy in ATTRv [36]. Grades on the Perugini scale, which is used to visually compare tracer uptake in the myocardium with that in the bones, range from 0 (no cardiac uptake and normal bone uptake) to 3 (strong cardiac uptake with little or no bone uptake); grades 2–3 indicate definite (ATTR-) CA. Although bone scintigraphy cannot reliably distinguish between subtypes of CA, mild uptake (grade 1) or absence of uptake suggests other forms, particularly AL-CA, which requires further evaluation. The mechanism behind the differential uptake in ATTR and AL is unknown. It has been proposed that greater calcium content in ATTR underlies its preferential uptake. The American Society of Nuclear Cardiology no longer recommends planar imaging-derived metrics alone for the diagnosis of ATTR-CM, mandating visual assessment using single-photon emission computed tomography (SPECT) [1]. SPECT enhances diagnostic accuracy by differentiating true myocardial uptake from blood pooling, especially in early disease or renal impairment.

In summary, if a 99mTc-PYP/DPD/HMDP scan is grade 2 or 3 and no monoclonal protein is detected in blood or urine (with serum/urine immunofixation and a serum FLC assay), ATTR cardiac amyloidosis can be diagnosed without biopsy. In patients with evidence of a plasma-cell dyscrasia, histological confirmation remains necessary [14].

#### 2.2.2. Bone Scintigraphy for Monitoring of Disease Progression

The suitability of bone scintigraphy for therapy monitoring in cardiac amyloidosis remains a matter of ongoing debate. One limitation is that evaluation using the Perugini classification is only semiquantitative and relatively imprecise. Moreover, recent studies suggest that the affinity of radionuclides (^99m^ Tc-phosphates) may change during disease progression, possibly due to structural alterations of the amyloid fibrils. On the other side, a study in untreated ATTR-CA patients who underwent serial ^99m^Tc bone scans over time demonstrated no relevant changes in H/CL ratios despite clinical evidence of disease progression [37].

In the phase 3 HELIOS-A trial evaluating vutrisiran in ATTRv amyloidosis, 99mTc scintigraphy was performed in 64 treated patients at baseline, with 55% showing Perugini grade ≥2 cardiac uptake. After 18 months, 28% demonstrated a reduction in grade, 68% remained unchanged, and 4% showed worsening [38]. The heart-to-vertebral ratio has been proposed as an alternative quantitative index that overcomes limitations of the heart-to-contralateral lung (H/CL) ratio, such as blood pool and rib uptake. In ATTR-ACT trial data, this ratio and visual scores remained stable in a placebo-treated patient over 30 months but improved after 45 months of tafamidis therapy [39].

**Figure 3:**
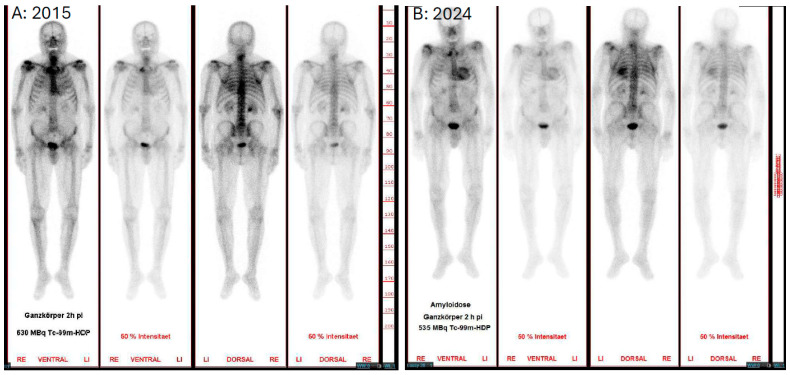
Late phase of a bone scintigraphy of the same person with a 9-year difference. (**A**): Bone scintigraphy in 2015 with the indication of searching for metastases in prostate cancer—no cardiac accumulation. (**B**): Bone scintigraphy in 2024 as part of amyloidosis screening—Perugini grade 3 uptake of the heart.

In general, however, serial SPECT ^99m^ Tc-PYP/DPD/HMDP scintigraphy does not appear to be a suitable tool for monitoring of therapy at this point in time with currently available treatment options [14]. Accordingly, the American Society of Nuclear Cardiology does not recommend serial nuclear imaging after an initial abnormal scan or for monitoring of progression or treatment response (2024), so this method should be reserved for research purposes only [40]. However, repeated bone scintigraphy may be considered if amyloidosis is suspected in the primary diagnosis and the first scan is negative due to a time lag (Figure 3).

### 2.3. Cardiac Magnetic Resonance Imaging

#### 2.3.1. CMR for Primary Diagnosis

Cardiac magnetic resonance imaging has become an indispensable tool, offering highly accurate and reproducible assessment of cardiac function, as well as noninvasive tissue characterization, that enables differentiation between various heart diseases, especially hypertrophic forms. Therefore, CMR has a wide range of Class I indications in multiple guidelines [2,41]. Cine imaging allows for detailed anatomical and functional evaluation of both ventricles and atria, including strain analysis similar to echocardiographic speckle tracking, especially in cases of poor echocardiographic windows. While CMR can also demonstrate the apical sparing pattern, its greater value lies in precise tissue characterization.

**Figure 4:**
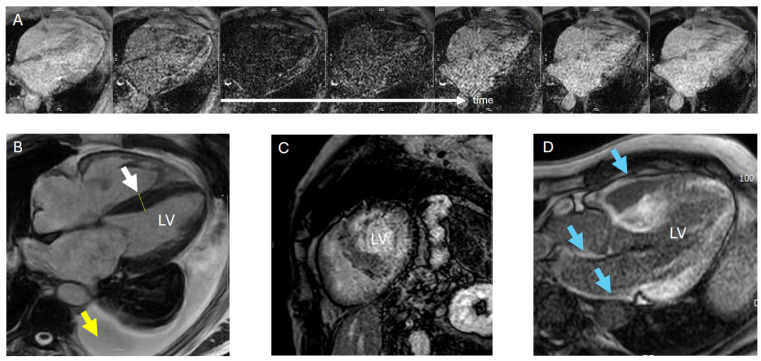
Typical cardiac magnetic resonance findings in amyloidosis. (**A**) Difficulty in achieving myocardial nullification in look-locker sequence/inversion-time scout. (**B**) SSFP cine four-chamber view with septal thickening and pleural effusion. (**C**) IR TFE sequence showing transmural LGE (late gadolinium enhancement) pattern, making it difficult to differentiate between myocardium and blood pool. (**D**) Typical LGE image in a three-chamber view, with contrast enhancement also visible in the atria and right ventricle. White arrow: septal thickening; yellow arrow: pleural effusion; blue arrow: LGE in the right ventricular wall, interatrial septum and atrial walls; LV: left ventricle.

LGE imaging detects structural myocardial abnormalities based on gadolinium contrast distribution. Cardiac amyloidosis shows a characteristic, almost pathognomonic enhancement pattern—beginning subendocardially and often appearing circumferential in cross-section—with a diagnostic sensitivity of about 95% and specificity of 98%. This is typically associated with “abnormal gadolinium kinetics”, whereby the gadolinium-enhanced myocardium and the blood pool are nullified at the same time, which makes it difficult for the operator to find the correct inversion time (Figure 4). Combined with the absence of monoclonal gammopathy, this provides a 98% positive predictive value for ATTR [8,25]. When gadolinium-enhanced CMR is likely to change management by confirming specific cardiomyopathy and enabling targeted therapy, its small nephrogenic systemic fibrosis risk is generally outweighed by the benefit of timely disease-modifying treatment.

**Figure 5:**
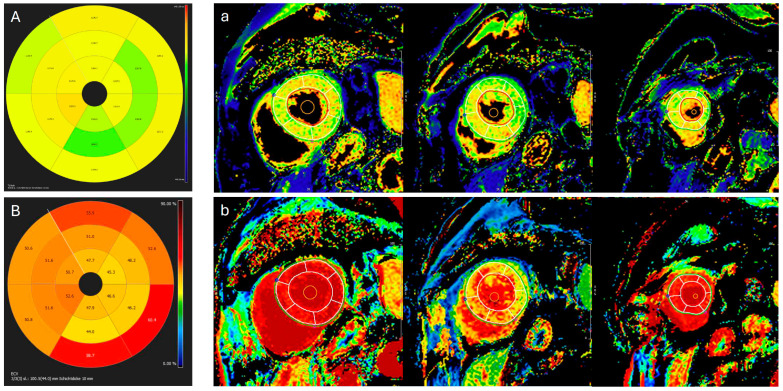
Cardiac magnetic resonance T1- and ECV (extracellular volume) maps in the short axis. (**A**) Bullseye map of native T1 mapping values (global value: 1163 ms; normal T1 range: 950–1050 ms). (**a**) Three associated short-axis native T1 maps with AHA (American Heart Association) segments. (**B**) Bullseye map of ECV mapping values (global value: 52%; normal ECV range: 25–31%). (**b**) Three associated short-axis native ECV maps with AHA segments.

Unlike LGE, T1 mapping provides an objective, quantitative measurement because it is a pixel-based reconstruction of measured longitudinal relaxation times, offering further improvements in myocardial tissue assessment. Native precontrast T1 is also prognostic but influenced by both intra- and extracellular changes. After gadolinium, combining pre- and post-contrast T1 values with the patient’s hematocrit isolates the extracellular signal and yields a quantitative extracellular volume (ECV) map (Figure 5). Normal global ECV is 25–30%, while values up to 70–80% indicate extensive amyloid infiltration. Color-coded T1 and ECV maps allow for rapid visualization of disease burden and can be used for primary diagnosis and the monitoring of progression or therapeutic response. As mentioned above, analogous to the differences in echocardiography strain, amyloid deposition within the myocardium is distributed heterogeneously. This means that disease-modifying therapies can affect ECV either globally or segmentally. The prognostic significance of this must be investigated in more detail. However, only extracellular volume (ECV) remains independently associated with mortality after adjustment for biomarker stage, indicating a more robust measure of disease burden because it directly quantifies amyloid deposition [42]. An interesting finding is the “native T1–ECV paradox”, with lower native T1 values despite higher myocardial mass and ECV in ATTRwt compared with AL. This may reflect a lower ratio of intracellular to extracellular myocardial components, which influences native T1 [43].

Because the T2 signal in CMR rises proportionally more than T1 with increased free water, it is well suited to identify edema, and quantitative T2 mapping can help track inflammatory diseases. Pathophysiologically, cardiac AL differs from ATTR, involving not only extracellular light-chain fibril deposition but also direct cytotoxic effects that induce myocardial inflammation and edema. Consequently, active cardiac AL is expected to show elevated native T2 values relative to ATTRwt. There are differing study results on this issue, so further multicenter studies are needed to clarify these discrepancies [43,44,45].

While the new mapping approaches are very helpful, it must be acknowledged that evidence on precision and reproducibility is still limited, particularly regarding physiological influences and differences between global and segmental measurements. Overall, our own scan–rescan approach suggests that absolute global changes >28 ms (or >3%) in T1 and >2 ms (or >4%) in T2 are indicative of relevant intra-individual inter-study variation and cannot be explained solely by random measurement noise or normal biological variability in healthy people [46]. In addition, mapping values are affected by physical factors (field strength and temperature) and methodological choices (sequence type, recovery timing/heart-rate dependence, and flip angle/SNR), so values can vary systematically beyond true biology. There are also subtle differences in T1 and T2 that are related to gender and age [47]. These effects are minor in large-change diseases (e.g., amyloidosis) but matter when assessing subtle processes like diffuse myocardial fibrosis. It may be concluded that subtle, preclinical changes are potentially more prone to error and more difficult to interpret than it is to monitor the progression of clearly pathological values.

Several international scientific societies have published position statements, each proposing slightly different diagnostic algorithms. All emphasize the role of cardiovascular magnetic resonance in the detection of cardiac involvement; however, the statement from the German Cardiac Society (DGK) explicitly incorporates a CMR-based pathway into the diagnostic algorithm, placing it on an equal level with myocardial scintigraphy [8]. The European Society of Cardiology (ESC) position statement indicates CMR and echocardiography as equivalent tools for the diagnosis of CA [5]. The recommendation in the diagnostic guidelines for CA in Asia [48] goes even further and serves CMR as an initial work-up screening modality (analogous to the Class 1 recommendation in patients with unexplained left ventricular hypertrophy in the ESC cardiomyopathy guidelines [2]) before bone scintigraphy.

In the consensus document of Dorbala et al. [14,15], CMR findings fall into three categories:

1. Not suggestive: Normal LV wall thickness, normal LV mass, no ventricular LGE, and normal atrial size;
2. Strongly suggestive: Increased LV wall thickness, increased LV mass, biatrial enlargement, typical diffuse or global LGE pattern, difficulty in achieving myocardial nullification, significantly increased global ECV (>0.40), and/or small pericardial and or pleural effusions;
3. Equivocal: Findings not described above.

#### 2.3.2. CMR for Monitoring Disease Progression

Accumulating evidence suggests that interval CMR imaging may ultimately play an important role in long-term disease monitoring. Cine CMR provides superior reproducibility and accuracy for assessment of LV geometry and function compared with echocardiography [6]. This makes it particularly well suited to ensure the reproducibility of follow-up data.

Transmural LGE was found to be predictive of death in both ATTR-CA and AL-CA but with a shorter median survival in patients with AL-CA [49]. As mentioned above, the use of CMR-derived ECV as a marker of amyloid burden is a significant advancement. Of note, ECV serves as a surrogate for amyloid burden and is not a direct measure of amyloid load. Serial ECV assessments may allow for direct visualization of fibril removal and disease regression after treatment. Increased ECV was found to be associated with reduced prognosis in ATTR-CA [50], and post-treatment reduction in ECV has been identified as a prognostic marker in AL-CA in several studies [51,52]. Additionally, with routine CMR, hepatic and splenic ECV can be mapped accurately, quantifying extra-cardiac amyloid burden and possibly tracking therapeutic effects, without added sequences [53]. T_2_ levels were found to be raised in untreated AL-CA compared with ATTR-CA and were predictive of prognosis in AL-CA only [44].

Similar to the echo data of individuals treated with tafamidis, the CMR-measured left ventricular (LV) ejection fraction (LV-EF), LV mass index, LV wall thickness, native T1 and extracellular volume (ECV) remained stable over 12 months of treatment [54]. Reduced ECV (along with a reduction in uptake on DPD) was observed 12 months after treatment with patisiran therapy in ATTR-CA [55]. The ATTRIBUTE subanalysis by Razvi et al. in 2024 was the first longitudinal CMR evaluation included within a phase 3 ATTR-CM trial [56], but findings were reported only descriptively due to the small sample size.

However, interpretation of mapping data should be approached with caution until prospective longitudinal studies supported by histological correlation provide stronger evidence to validate current assumptions and clarify the significance of noninvasive multiparametric CMR findings [45].

### 2.4. Other Imaging Modalities

Because current evidence is based on small, mostly retrospective studies, the role of positron emission tomography (PET) in diagnosing cardiac amyloidosis remains uncertain. Tracers such as ^11^C-Pittsburgh Compound B (PIB), ^18^F-florbetaben, and ^18^F-florbetapir—originally developed for detection of β-amyloid in Alzheimer’s disease—can visualize both ATTR and AL deposits, with reported diagnostic sensitivities and specificities ranging from 87 to 100% and 83 to 100%, respectively. Radiation exposure typically ranges between 1.5 and 7 mSv [8,57].

The new imaging methods are very promising but still experimental and have only been evaluated in small cohorts, meaning that they are not currently recommended for use in standard diagnostic pathways.

For example, cardiac CT-derived extracellular volume (ECVCT) offers a potential alternative for assessing cardiac amyloidosis, especially where MRI is less accessible. In a study of 72 already diagnosed patients (35 AL and 37 ATTR), mean ECVCT was elevated—42.7% in AL and 55.8% in ATTR. Higher ECVCT values predicted mortality in ATTR but not AL [58]. Although still experimental, ECVCT may become a valuable tool for future disease monitoring.

Future research offers the opportunity to use PET for quantitative assessment and therapy monitoring. PIB-PET data suggest that myocardial uptake correlates with amyloid burden and outcomes in AL [59]. However, these are experimental methods without evidence; therefore, they are not included in the current consensus recommendations.

## 3. CA Staging Systems and Monitoring Modalities

Using standardized staging systems ensures consistent and objective evaluation across patients and institutions, allows for clear comparison of findings, supports reliable monitoring of disease course, and guides treatment decisions based on defined prognostic categories. Therefore, it is all the more surprising that the cardiac amyloidosis staging systems predominantly used in studies are not yet widely adopted in clinical practice and that crucial imaging criteria are largely overlooked. An explanation could be that NT-proBNP proved to be a more sensitive marker than conventional echocardiographic parameters for the detection of clinical improvement or deterioration in amyloid cardiomyopathy during follow-up in early studies [60]. However, the specificity of NT-pro BNP and troponin is low, and there are still no markers available to diagnose the disease in its preclinical stage. NT-proBNP reflects general myocardial stress and is affected by factors like renal dysfunction; therefore, its interpretation can be limited. There is evidence that an increase in TTR levels during treatment indicates a reduction in cardiovascular risk and may therefore be an independent predictor of survival. Despite these groundbreaking results, the findings on biomarkers are still incomplete. This review focuses on imaging parameters in the treatment of amyloidosis and is not intended to devalue laboratory parameters. These are easy and quick to measure, widely available, independent of the examiner’s skills and generally less expensive than large-scale imaging equipment. Therefore, only an integrative multimodal approach to progress monitoring can be effective.

Table 1 provides an overview of the most commonly used prognostic scores of the various staging systems. Recently, one research group published an additional imaging parameter (echocardiographic GLS) in hereditary ATTR amyloidosis [61], with GLS often having been shown to be prognostically significant in other studies [30]. Older and established staging systems for AL and ATTR use laboratory parameters exclusively [62,63,64,65,66]. Current guidelines define cardiac response to AL treatment primarily through NT-proBNP reduction, with a response indicated by >30% and >300 ng/L decrease from baseline. Frequent increases in NT-proBNP and diuretic intensification are consistently linked to higher mortality and, when combined, offer a simple, widely applicable model for detecting disease progression in ATTR-CA [67].

In 2021, Garcia-Pavia et al. published an expert consensus on the monitoring of ATTR cardiomyopathy [6]. Assessment of cardiac disease status should be incorporated into a comprehensive multiparametric framework that concurrently evaluates progression, stability, or improvement in extracardiac organ involvement. Within this context, eleven quantifiable variables spanning three domains were proposed: “1. clinical and functional endpoints, 2. circulating and laboratory biomarkers, and 3. imaging and electrocardiographic indices” [6]. The expert consensus determined that the inclusion of at least one representative parameter from each domain constitutes the minimum standard for a robust evaluation of disease progression.

**Figure 6:**
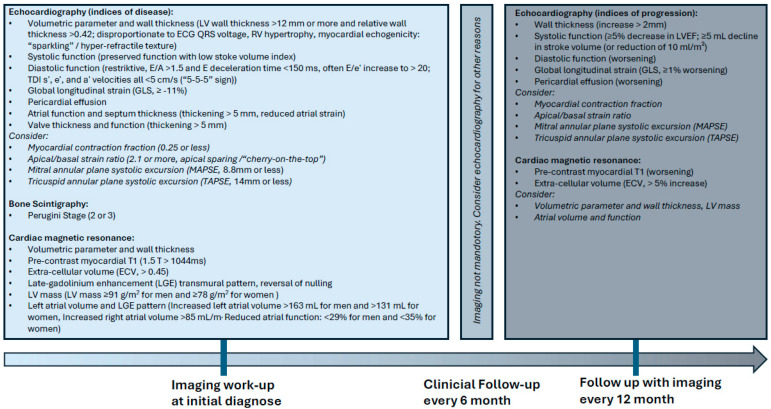
Proposed imaging parameters on the timeline. Most available evidence is based on small retrospective cohorts. The resulting conclusions have largely been shaped by expert appraisal and consensus recommendations rather than high-level prospective trial data. Normal font: generally recommended and frequently used parameters (comparable to Class I–IIa recommendations); italic font: recommendations that are less well documented (analogous to Class IIb recommendations).

Around the same time, Patel et al. specified the imaging parameters to be measured for cardiac amyloidosis based on available study results, although the proposed cut-off values still require further evaluation [68]. Deterioration of LV dimensions, stroke volume index, mitral E/e’, GLS and ECV (measured every 6 months) was defined as indicating disease progression, and improvements in GLS, the apical/base strain ratio, LV mass and ECV (measured every 6 months), as well as improvements in septal wall thickness and LVEF (measured every 3 years), were defined as indicating disease regression. Modified, consensus-based measurement instruments for the longitudinal evaluation of disease progression are listed in Table 2. On this basis and taking into account the above-mentioned evidence-based imaging parameters, as well as economic and clinically relevant aspects, we propose a simple, literature-derived algorithm for multimodal imaging for evaluation of CA disease progression in Figure 6. The diagram shows a summary of the primary studies mentioned in the text; the already published secondary literature on the guidelines, which themselves reflect expert opinions [6,14,15,68]; and our own experience, with an emphasis on published MRI data, which has been under-represented to date.

Table 2 presents data based on the work of Garcia-Pavia et al., with modifications and a focus on imaging parameters. New content based on evaluated parameters of other research groups is presented in italics—mainly Patel et al. and ASNC/AHA/ASE/EANM/HFSA/ISA/SCMR/SNMMI Expert Consensus Recommendations for Multimodality Imaging in Cardiac Amyloidosis.

## 4. Current Therapeutic Agents and the Role of Imaging Parameters

Historically, solid-organ transplant was the only option for ATTR. Cardiac transplantation was rarely feasible in ATTRwt due to advanced patient age, and in ATTRv, liver transplantation was used to remove mutant TTR but was limited by ongoing progression of cardiac amyloidosis and polyneuropathy after surgery [68].

Current AL therapies target plasma cells to lower free light chains, mainly using myeloma-derived regimens. ATTR-CA therapies include stabilization of TTR tetramer and RNAi TTR gene silencing. Specific therapies complement guideline-based heart failure therapy. An overview of current specific therapeutic agents is provided in Table 3, with a focus on the role of imaging in the approval studies [33,56,69,70,71,72]. It should be noted that despite the prominent role of imaging in diagnostics and its dramatically increasing importance in progress diagnostics, no approval study has included imaging parameters in the primary endpoints.

Further drugs are currently being tested. At the time of submission (October 2025) patisiran (Onpattro, a small interfering RNA (siRNA) that specifically degrades TTR messenger RNA (mRNA) to lower TTR protein production) is not approved for ATTR cardiomyopathy—approval remains limited to hATTR polyneuropathy, with the same status in the USA and the EU. Inotersen (Tegsedi) is likewise not approved for ATTR cardiomyopathy and is indicated only for hATTR polyneuropathy. Diflunisal (nonsteroidal anti-inflammatory drug acting as a TTR stabilizer; approval recommendation for the treatment of ATTRv adult patients with stage 1 or 2 polyneuropathy) has no regulatory approval for ATTR cardiomyopathy; it is sometimes used off-label as a TTR stabilizer, supported mainly by observational and meta-analytic data but limited by adverse effects and discontinuations [73].

The following simple rule applies to all measurement methods, whether laboratory or imaging methods: The method must be suitable for the intended purpose. It can be said that the pharmacological effect of current therapies is not aimed at reducing the existing amyloid burden but at preventing progression. Therefore, from a pathophysiological point of view, an improvement in the existing imaging parameters is not to be expected at all. Therefore, a lack of improvement in the imaging parameters in most of the presented studies cannot be regarded as a failure of therapy. The imaging endpoints of current drug trials should be deliberately defined as ‘no change’, since any improvement cannot be explained as a pharmacological drug effect but is presumably due to deeper processes such as the body’s own autoimmune response. In contrast to existing therapies, future depleters are expected to show a clear regression of the imaging parameters. The definitions for progress are listed in Table 2 and Figure 6, insofar as they are known and have been evaluated in studies. Another aspect to consider is that measurement variability must be taken into account. To minimize methodological inaccuracies, it is recommended that a follow-up examination be carried out at the same center using the same device and, if possible, by the same examiner. In summary, the value of imaging currently lies only in monitoring a lack of progression, but it will become apparent as an improvement in future amyloid-reducing therapies.

## 5. Limitations and Further Perspectives

With growing insight into the pathophysiology of cardiac amyloidosis, new therapeutic targets are emerging [74]. Earlier diagnosis through improved imaging has enhanced survival [75]. However, rising trial costs highlight the need to explore imaging markers as potential surrogates for treatment efficacy.

Risk stratification is essential to tailor therapy and follow-up to the anticipated disease trajectory. Although standardized scoring systems exist, most lack integration of imaging parameters, and their application in cardiac amyloidosis remains undefined. Evidence from CMR and nuclear imaging studies indicates that inhibition of further amyloid deposition may reduce cardiac amyloid burden and that imaging can be used to monitor response to disease-modifying therapies. Current evidence is dominated by small, single-center series. The central challenge of imaging in amyloidosis in clinical practice is the still limited integration of imaging findings into treatment decision-making. To date it remains unclear when an imaging result should actually trigger a change in therapy, how to interpret imaging progression when biomarkers remain stable, and what should be considered a truly discordant response between imaging and biomarker trajectories. It is also unresolved as to whether mere stabilization is an adequate treatment target. At present, there are no robust data that provide evidence-based answers to these questions. Importantly, multimodal imaging findings indicative of disease progression should not be interpreted as justification for discontinuing disease-modifying therapy. Therefore, it is essential to encourage prospective studies and, in parallel, to systematically collect data and experience in clinical routine to enable well-founded retrospective conclusions.

In future intervention trials with respect to ATTR and AL, echocardiographic and cMR imaging should be included, adhering to the diagnostic standards published in consensus documents. Time intervals for follow-up studies should also be standardized to permit later pooling of phase III imaging substudies. In parallel, validation of imaging biomarkers, including CMR-based T1 mapping and ECV quantification, as well as radionuclide techniques such as SPECT and PET, is essential to establish reliable measures of CA progression.

ATTR-CM usually progresses with ongoing amyloid deposition, and no therapy has been shown to reverse the condition to date. Fontana et al. reported three men with spontaneous resolution of ATTR-CM-related heart failure, despite not receiving disease-modifying therapy. This recovery was supported by improvements in the clinical course, as well as in laboratory and multimodal imaging results (near-complete regression of ECV in CMR, remodeling to near-normal cardiac structure and function without scarring, and DPD scintigraphy showing a decrease in cardiac tracer uptake). One biopsy showed unusual inflammation around the amyloid, and all three patients had high-titer anti-ATTR IgG antibodies of uncertain significance, which suggests rare reversibility and a potential immunological mechanism [76]. Therefore, a recombinant human anti-ATTR antibody that removes ATTR by activating phagocytic immune cells could potentially lead to a genuine cure and has already been tested in a phase 1 trial. Cardiac tracer uptake on scintigraphy and ECV on CMR were used for follow-up and appeared to reduce over a period of 12 months [77].

Since amyloidosis is difficult to diagnose due to the varied and multiple presentation of findings, studies have already been conducted to train artificial intelligence systems using information from amyloidosis patients. Previous studies have primarily trained AI using data from patients who already have a clinical diagnosis of cardiac amyloidosis, often with advanced disease. When such models are used for screening in the general, asymptomatic population, sensitivity decreases significantly or the presence of subtle or mild abnormalities may yield misleading or false-positive results. New AI methods could reduce these limitations, e.g., methods that predict continuous rather than categorical values [10]. This opens up another field of research.

## 6. Conclusions

Advances in multiparametric cardiac imaging—combining echocardiography, bone scintigraphy and cardiac MRI—have enabled earlier diagnosis and treatment of all forms of cardiac amyloidosis. Interestingly, there are no standardized imaging methods for assessment of its progression to date. One argument for this is that current therapeutic approaches are not designed to reduce amyloid burden, so it is unlikely that imaging would show any improvement in parameters—only stabilization. Nevertheless, there is growing evidence that follow-up examinations using echocardiography, in particular, and CMR with techniques such as longitudinal strain and extracellular volume quantification can provide valuable information about changes to the heart that occur in response to the disease’s natural progression or drug treatment. In addition, such an approach can also reveal structural and functional abnormalities that influence the underlying disease, making it valuable for basic research.

## Figures and Tables

**Table 1:** Selection of common staging systems for cardiac amyloidosis (AL and ATTR).

Disease/System	Parameters	Stage	Criteria/Thresholds	Reference
AL Amyloidosis—Mayo 2004	NT-proBNP, Troponin T	I	Both below threshold (TnT < 0.035 µg/L, NT-proBNP < 332 ng/L)	Dispenzieri et al., J Clin Oncol, 2004 [65]
		II	One marker above threshold	
		III	Both markers above threshold	
AL Amyloidosis—European modification	NT-proBNP, Troponin T	IIIa	Mayo stage III and NT-proBNP ≤ 8500 ng/L	Wechalekar et al., Blood, 2013 [66]
		IIIb	Mayo stage III and NT-proBNP > 8500 ng/L	
AL Amyloidosis—Revised Mayo (Kumar, 2012)	NT-proBNP, Troponin T, difference between involved and uninvolved light chain (dFLC)	I	Score of 1 for each: FLC-diff ≥ 18 mg/dL, cTnT ≥ 0.025 ng/mL, and NT-ProBNP ≥ 1800 pg/mL, no marker elevated	Kumar et al., J Clin Oncol 2012 [62]
		II	1 marker elevated	
		III	2 markers elevated	
		IV	3 markers elevated	
ATTRwt and ATTRv Amyloidosis—NAC (Gillmore, 2017)	NT-proBNP, eGFR	I	NT-proBNP ≤ 3000 ng/L & eGFR ≥ 45 mL/min/1.73 m^2^	Gillmore et al., Eur Heart J. 2018 [64]
		II	One parameter abnormal	
		III	NT-proBNP > 3000 ng/L and eGFR < 45	
ATTRwt Amyloidosis—Mayo (Grogan, 2016)	NT-proBNP, Troponin T	I	Both below threshold (TnT < 0.05 ng/mL, NT-proBNP < 3000 ng/L)	Grogan et al., J Am Coll Cardiol. 2016 [63]
		II	One marker above threshold	
		III	Both markers above threshold	
ATTRv Amyloidosis—(Neculae, 2025)	NT-proBNP, eGFR, Global Longitudinal Strain (GLS)	I	No criteria present (GLS < −11%, NT-proBNP < 2000 ng/L, eGFR > 65 mL/min)	Neculae et al., ESC Heart Fail 2025 [61]
		II	Intermediate: not meeting Stage I or III criteria	
		III	GLS ≥ −11% and either NT-proBNP ≥ 2000 ng/L or eGFR ≤ 65 mL/min	

**Table 2:** Recommended measurement tools for detecting disease progression in patients with amyloid cardiomyopathy.

Domain	Tool/Clinical Feature	Threshold Indicating Disease Progression	Recommended Frequency
Clinical and Functional	Cardiovascular hospitalizations	Hospitalization for heart failure decompensation	Every 6 months
	NYHA functional class	One-class increase	Every 6 months
	Quality of life (EQ-5D, KCCQ)	≥5-point decrease = deterioration	Every 6–12 months
	6-Minute Walk Test (6MWT)	Decline of 30–40 m over 6 months	Every 6 months
Biomarkers	NT-proBNP	≥30% increase with absolute rise ≥300 pg/mL	Every 6 months
	High-sensitivity troponin	≥30% increase from baseline	Every 6 months
	Clinical staging (NAC score)	Advancement NAC stage	Every 6 months
Imaging	Echocardiography—LV wall thickness/mass	≥2 mm increase in LV wall thickness	Every 6–12 months
	Echocardiography—systolic function	≥5% decrease in LVEF; ≥5 mL decline in stroke volume *(or reduction of 10 mL/m*^2^*)*; ≥1% worsening in LV global longitudinal strain *or GLS ≥ −11%* *Myocardial contraction fraction of 0.25 or less* *Apical/basal strain ratio of 2.1 or more* *Mitral annular plane systolic excursion (MAPSE) of 8.8 mm or less* *Tricuspid annular-plane systolic excursion (TAPSE) of 14 mm or less*	Every 12 months
	Echocardiography—diastolic function	Stepwise worsening of diastolic function grade *(using inferior vena cava diameter or Doppler assessment including E/e’ [increase to > 20] and transmitral flow), pericardial effusion*	Every 12 months
	*Magnetic resonance imaging*	*Pre-contrast myocardial T1* *(1.5 T field strength; using shMOLLI) > 1044 ms* *Extracellular volume (ECV) > 0.45 or >5% increase* *Late-gadolinium enhancement (LGE) transmural pattern* *LV mass ≥91 g/m*^2^ *for men and ≥78 g/m*^2^ *for women (with papillary muscle included)* *Increased left atrial volume >163 mL for men and >131 mL for women, increased right atrial volume >85 mL/m*^2^, *reduced atrial function: <29% for men and <35% for women*	
ECG	ECG/Holter ECG	New-onset arrhythmia or conduction abnormality	Every 6 months

**Table 3:** Role of imaging parameters used in Phase III studies of approved therapies.

Indication	Therapy (Brand) & Phase III Trial Name	Mechanism	Study Endpoints	Role of Echocardiography (Published Data)	Role of CMR (Published Data)	Reference
AL (systemic, incl. cardiac)	Daratumumab + CyBorD (DARZALEX FASPRO^®^ + bortezomib/cyclophosphamide/ dexamethasone) ANDROMEDA	CD38 monoclonal antibody (plasma cell–directed)	Hematologic complete response rate Cardiac involvement/response adjudicated primarily via biomarkers (Response Progression Heart: NT-proBNP response (>30% and >300 ng/L decrease in patients with baseline NT-proBNP ≥650 ng/L) or NYHA class response (≥2 class decrease in patients with baseline NYHA class 3 or 4) NT-proBNP progression (>30% and >300 ng/L increase) or cTn progression (≥33% increase)	Ejection fraction criterion of progression (≥10% decrease) No primary endpoint data	No CMR data	Kastritis et al. [69]
ATTR-CM (wild-type & variant)	Tafamidis (Vyndaqel^®^/ Vyndamax^®^) ATTR-ACT	TTR tetramer stabilizer	Hierarchical composite: all-cause mortality & CV hospitalizations; secondary endpoints were the change in 6 min walk test and KCCQ-OS over 30 months	Inclusion required septal thickness >12 mm on echocardiography. Echo analyses tracked LVEF, LV stroke volume, global longitudinal strain (GLS), radial strain, and wall thickness over 30 months. Smaller decreases in left ventricular stroke volume were noted at month 30. No primary endpoint data ATTR-ACT Echo-subanalysis: Over 30 months, there was less pronounced worsening in 4 of the echocardiographic measures; LV stroke volume, 7.02 mL; LV global longitudinal strain, −1.02%; septal E/e’, −3.11 and lateral E/e’, −2.35 in tafamidis treated patients vs. placebo	No CMR data	Maurer et al. [33] Shah et al. [70]
ATTR-CM (wild-type & variant)	Acoramidis (Attruby™/ Beyonttra™) ATTRIBUTE-CM	Next- generation TTR stabilizer	Four-component hierarchical: all-cause mortality, CV hospitalizations, NT-proBNP, 6 min walk distance; secondary outcomes were death from any cause, the 6 min walk distance, the score on KCCQ-OS, and the serum TTR level.	Eligibility included LV wall thickness ≥12 mm by echocardiography. No primary endpoint data.	CMR-subanalysis: LVEF, LV SVi, GLS, LV mass, ECV, RV-EF, RV SVi, myocardial contraction fraction and ECV (amyloid regression was defined as an absolute reduction in ECV ≥5%, progression as an absolute increase in ECV ≥5%) were measured. Findings are reported descriptively due to the small sample size.	Gillmore et al. [71] Razvi et al. [56]
ATTR-CM (wild-type & variant)	Vutrisiran (AMVUTTRA^®^) HELIOS-B	RNAi TTR silencer	Composite of all-cause mortality + recurrent CV events (CV hospitalizations and urgent HF visits) at 30–36 mo; secondary endpoints included death from any cause, change in the 6 min walk test, and the change in KCCQ-OS Exploratory endpoints of NT-proBNP level, troponin I level, peak longitudinal strain, and EuroQol 5-Dimension 5-Level questionnaire	Interventricular septal wall thickness exceeding 12 mm in echo for inclusion. Exploratory endpoint: Peak longitudinal strain change from baseline at 30 months (LS mean (SEM) Vutisiran 0.95 (0.17) vs. placebo 2.18 (0.19), LS mean difference −1.23 (95% CI −1.73, −0.73)	No published CMR data	Fontana et al. [72]

## Data Availability

No new data were created or analyzed in this study.
